# Progress in and Opportunities for Applying Information Theory to Computational Biology and Bioinformatics

**DOI:** 10.3390/e24070925

**Published:** 2022-07-03

**Authors:** Alon Bartal, Kathleen M. Jagodnik

**Affiliations:** The School of Business Administration, Bar-Ilan University, Ramat Gan 5290002, Israel; kathleen.jagodnik@biu.ac.il

This editorial is intended to provide a brief history of the application of Information Theory to the fields of Computational Biology and Bioinformatics; to succinctly summarize the current state of associated research, and open challenges; and to describe the scope of the invited content for this Special Issue of the journal *Entropy* with the theme of “Information Theory in Computational Biology”.

Information Theory as a field of research was established with the publication of Claude Shannon’s seminal monograph “A Mathematical Theory of Communication” in 1948 [1]. This work introduced concepts including information entropy, mutual information (a term that was later coined by Roberto M. Fano [2]), and the representation of information as binary digits (bits, a term that is credited to John Tukey) [3]. Progressing beyond earlier related work by Harry Nyquist and Ralph Hartley in the 1920s, and by Alan Turing and Norbert Wiener in the 1940s [4,5], Shannon’s work describes the fundamental laws of data transmission and compression [6] and the theoretical limits on the efficiency of communicating over noisy channels [7]. As a unifying theory that intersects with many disciplines including Probability, Statistics, and Computer Science [6], Information Theory is applied to study the extraction, transmission, processing, and use of information in a variety of systems. Shannon’s concepts, and those inspired by them, underlie modern digital information technology [5].

In the 1960s, improvements in experimental methods, including crystallography, and the rapid expansion of molecular biology methods across the biological subdisciplines, permitted biologists to advance our understanding of a variety of phenomena [8] including the characteristics of the RNA code [9], the structures of proteins [10,11], and the evolution of genes and proteins [10,12,13,14]. The central dogma of molecular biology [15] was developed following the foundational discoveries of the processes of RNA transcription and translation. With the advent of Computer Science theory and the era of modern computation starting in the 1960s, the application of computational strategies to address biological questions introduced the field of Computational Biology [16]. The early achievements in the application of computational methods to biological questions include computational studies of evolution [17] and protein structures [18], and the development of the first sequence alignment algorithms [19,20].

We note that Computational Biology is sometimes referenced interchangeably with Bioinformatics [21,22,23], although these disciplines are also often differentiated in various ways. We make the following distinction: Bioinformatics seeks to develop algorithms, databases, software tools, and other computational resources that permit the insightful analysis of biological data, including its acquisition, storage, quantification, annotation, visual exploration, and other forms of processing [23]. A single software-based product of a Bioinformatics project can often be widely applied to address a variety of biological questions. Complementing the scope of Bioinformatics, Computational Biology seeks to answer specific biological questions using computational strategies [23]. Some Computational Biology projects develop algorithms and computational tools to analyze biological data for addressing the question of interest, and many Computational Biology analyses use the tools created by bioinformaticians. The work of many researchers spans both domains. Both of these disciplines have benefited from the application of Information Theory, and accordingly, this Special Issue welcomes submissions involving the application of Information Theory to both Computational Biology and Bioinformatics.

Information Theory has been among the strategies used to advance Computational Biology from the earliest period of the latter discipline’s development onward. Initial studies included reports on the informational properties of DNA [24] and protein sequences [11]. Information Theory has been applied at the molecular level to quantify information of DNA binding sites [25], to understand gene regulation and metabolic networks [26], and to study protein–DNA interactions [25] and protein–protein interactions [27]. It has also been used to elucidate biological sequences [28]. Information Theoretical concepts such as Mutual Information have been employed for protein structure prediction, including the identification of co-evolving amino acid residues [29,30,31,32,33,34]. Signal transmission in cellular systems, which are inherently noisy, has been quantified using Information Theoretical concepts including entropy [27,35,36]. Information Theory has facilitated the effective modeling of non-linear relationships involving biological entities and has contributed to representing biological systems as stochastic processes [37]. This brief editorial only touches on the numerous applications of Information Theory to the fields of Computational Biology and Bioinformatics.

Since the 1990s, significant improvements in sequencing technology, and steady increases in computing power and reductions in the costs of computing, have led to an exponential increase in the generation of biological data [8]. The present era of ‘Big Data’ requires innovative strategies for data mining, exploration, and management [8], which can be addressed with Information Theory-based strategies. For example, while dimensionality reduction for omics datasets has often used Principal Components Analysis (PCA) [38], an alternative, Information Theory-based method, Independent Component Analysis (ICA), enables the identification of meaningful content using the measure of negentropy [37,39]. The ICA method has the advantage, relative to PCA, that it does not require latent factors to be orthogonal [37]. Additionally, managing massive quantities of biological data has been proposed via the entropy-scaling search, which was shown to dramatically accelerate searches of protein, metagenomic, and chemical data [40,41]. Many opportunities remain to apply Information Theory principles to further improve the management and effective use of biological data.

Recent innovations in Computational Biology and Bioinformatics invite new applications of Information Theory to these disciplines. For example, advances in analyses at the level of single cells have revolutionized many biological fields, as these techniques permit the high-resolution discovery of characteristics that are masked by bulk sampling strategies. Recent developments have started to apply Information Theory to the analysis of single-cell data, including gene expression data. Since single-cell gene expression data is characterized by distinctly different data distribution patterns and other properties [37], compared with bulk samples, new statistical methods are needed, for which Information Theory can be useful. As an example, Chan et al. [42] presented an approach to analyze single-cell gene expression data based on multivariate Information Theory, a strategy that is reported to be more reliable than classical approaches [43]. Additionally, entropy has been used to measure variability and other properties including stemness in single-cell transcriptomic data [44]. However, a significant need remains for more accurate and optimized analysis methods that are specific to single-cell data [37].

A variety of other promising potential applications of Information Theory to Computational Biology and Bioinformatics remain. Multi-omics integration is a strategy that continues to evolve, as new experimental platforms and data types emerge. Information Theory has previously been demonstrated as a useful approach for such an integration [45], and many opportunities remain to apply these concepts to yield more informative integrative analyses. More broadly, high-dimensional statistical theory for biological applications remains to be advanced, with a need for unifying definitions and interpretations of statistical interactions [37]. Information Theoretical concepts including entropy have facilitated the analysis of complex networks [46], which characterize a variety of biological systems [47,48]. The origins of life remain uncertain, and Information Theory has been demonstrated as a useful tool to address this problem [49]. The complex dynamics of neural information processing remain to be fully elucidated, and Information Theory has previously been applied to address these questions [50,51]. Assessing human physiological and emotional states in more informative and accurate ways can be facilitated with Information Theory concepts, including mutual information [52]. Shannon’s classical Information Theory is being advanced toward the use of quantum Information Theory [37] for applications including studying quantum information transfer from DNA to proteins [53], quantum-mechanical modeling of mutations in cancer [54], and error-correction coding in genetics [55]. This editorial provides a brief overview of some key opportunities for advancing Computational Biology and Bioinformatics by applying Information Theory; a more comprehensive review of the progress and open challenges is available in [37].

**The goal of this *Entropy* Special Issue** is to present a curated collection of expert perspectives on applying Information Theory to Computational Biology and Bioinformatics in diverse contexts. Its areas of research may include, but are not limited to, sequencing, sequence comparison, and error correction; gene expression and transcriptomics; biological networks; omics analyses; genome-wide disease-gene association mapping; and protein sequence, structure, and interaction analysis. Original research manuscripts, review papers, and Perspective/Commentary articles are welcome. The fields of Computational Biology and Bioinformatics, facilitated by continuing improvements in technology, can be advanced in exciting new directions by the thoughtful application of Information Theory principles.
